# Is conversion therapy possible in stage IV gastric cancer: the proposal of new biological categories of classification

**DOI:** 10.1007/s10120-015-0575-z

**Published:** 2015-12-07

**Authors:** Kazuhiro Yoshida, Kazuya Yamaguchi, Naoki Okumura, Toshiyuki Tanahashi, Yasuhiro Kodera

**Affiliations:** Department of Surgical Oncology, Gifu University Graduate School of Medicine, 1-1 Yanagido, Gifu, 501-1194 Japan; Department of Gastroenterological Surgery, Nagoya University Graduate School of Medicine, Nagoya, Japan

**Keywords:** Gastric cancer, Conversion therapy, Adjuvant surgery, Chemotherapy, Stage IV gastric cancer

## Abstract

Conversion therapy for gastric cancer (GC) has been the subject of much recent attention. It is defined as a surgical treatment aiming at an R0 resection after chemotherapy for tumors that were originally unresectable or marginally resectable for technical and/or oncological reasons. However, the indications for resection remain to be clarified. In the present review, we focus on the biology and heterogeneous characteristics of stage IV GC and propose new categories of classification. Stage IV GC patients can be divided based on the absence (categories 1 and 2) or presence (categories 3 and 4) of macroscopically detectable peritoneal dissemination, which has a different biological outcome compared to hematological metastasis. Category 1 is defined oncologically as stage IV but the metastasis is technically resectable. Category 2 includes a marginally resectable metastasis or patients for whom the operation would not necessarily be the best choice. Category 3 includes a potentially unresectable metastasis of peritoneal dissemination that is only macroscopically detectable. Category 4 includes noncurable metastasis with peritoneal and other organ metastasis. The indications for conversion therapy might include the patients from category 2, some patients from category 3 and a very small number of patients from category 4. The longer survival can be expected for patients corresponding to categories 1, 2 and, to a lesser extent, 3, while the treatment of other patients focuses on “care.” The provision of conversion therapy for stage IV GC patients might be one of the main roles of surgical oncologists in the near future.

## Introduction

In spite of early diagnosis and improved intensive treatments, GC remains the second leading cause of death throughout the world [1]. With the development of therapeutic approaches, the standard treatment of GC has been established, as is demonstrated in the Japanese treatment guidelines for GC [2]. Treatments include ESD in T1a tumors, laparoscopic surgery or, to a lesser extent, lymphadenectomy in T1b tumors and standard D2 lymphadenectomy for locally advanced resectable gastric cancer. As for metastatic and recurrent GC, several new regimens have been developed that improve patient survival [3–7]. However, the median survival time (MST) remains at only 13–16 months. In order to further improve the survival of stage IV GC patients, new therapeutic approaches should be considered [8, 9].

On the other hand, recent improvements in the survival of metastatic colorectal cancer patients have mainly depended on new cytotoxic and molecular target agents (FOLFOX, FOLFIRI, XELOX and SOX with bevacizumab, cetuximab, panitumab, regorafinib and ramcirumab), which have recently achieved an increase in MST from 6 to 30 months [10–17]. Moreover, the surgical approach to metastatic lesions designated for conversion therapy or an oncosurgical approach has played a very crucial role in the prolonged survival of metastatic colorectal patients [18–20] because this operation, which aims at R0 resection, might be able to cure patients with metastatic lesions for whom the major treatment would previously have been chemotherapy.

Although the prognosis of stage IV GC has recently improved as the result of new chemotherapeutic and molecular targeting agents, it remains unsatisfactory. With the development and improved response of the chemotherapy regimens, a number of conversion therapy approaches have been successfully demonstrated in stage IV GC [21–26]. However, significant aspects of these approaches, such as the indications, chemotherapy regimens and timing of the operation, remain to be clarified. In the present review, current developments in the treatment of stage IV GC will be discussed, conversion therapy will be defined and new categories of classification for stage IV GC will be proposed.

## The history of metastatic GC clinical trials in Japan

**Fig. 1:**
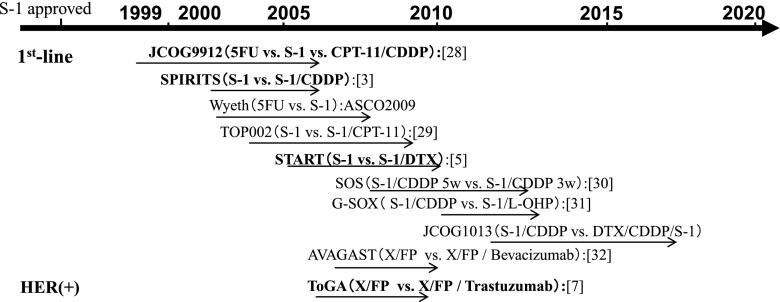
The history of metastatic GC clinical trials in Japan

According to the updated Japanese guidelines on gastric cancer [27], S-1 plus cisplatin (S-1/CDDP) is regarded as the first-line chemotherapy and is the first-level recommendation in HER 2-negative patients. The capecitabine plus cisplatin (XP) regimen and S-1 plus docetaxel (S-1/docetaxel) are also nominated as the second-level recommendations. On the other hand, in HER 2-positive patients, trastuzumab + XP is regarded as the recommended treatment regimen [7]. This consensus has been established on the basis of the history of clinical trials (Fig. 1), and the results of trials in Japan are summarized in Table 1. Trials in Western countries are also summarized in Table 2.

**Table 1:** Involvement of non-measurable lesions in Japanese clinical trials

Trial [Ref no.]	Treatment	Response rate (%)	Median overall survival (*M*)			Frequency of non-measurable lesions	
			Total population	Measurable	Non-measurable	(%)	Total
JCO9912 [28]	5-FU	9	10.8	N/A	N/A	37.2 % (87/234)	33.0 % (232/704)
	CPT-CDDP	38	12.3			32.2 % (76/236)	
	S-1	28	11.4			29.4 % (69/234)	
SPIRITS [3]	S-1	31	11.0	N/A	N/A	24 % (36/150)	29.2 % (87/298)
	S-1/CDDP	54	13.0			34 % (51/148)	
TOP-002 [29]	S-1	26.9	10.5	N/A	N/A	N/A	33.3 % (105/315)
	S-1/CPT	41.5	12.8			N/A	
START [5]	S-1	26.8	10.8	10.3	12.0	40.8 % (131/321)	39.3 % (250/635
	S-1/DTX	38.8	12.5	11.7	17.9	37.9 % (119/314)	
SOS [30]	SP5	50	13.9	N/A	N/A	37 % (113/309)	N/A
	SP3	60	14.1			39 % (120/306)	
G-SOX [31]	SP	52.2	13.1	N/A	N/A	19.8 % (64/324)	19.5 % (125/642)
	SOX	55.7	14.1			19.2 % (25/294)	
ToGA [7]	XP	35	11.1	N/A	N/A	11.3 % (33/290)	9.9 % (58/584)
	XP + Tmab	47	13.8			8.5 % (25/294)	
AVAGAST [32]	XP	37.4	10.1	N/A	N/A	23.2 % (90/387)	21.4 % (166/774)
	XP + Bev	46.0	12.1			19.6 % (76/387)

**Table 2:** Involvement of non-measurable lesions clinical trials of Western countries

Trial [Ref no.]	Treatment	Response rate (%)	Median overall survival (*M*)			Frequency of non-measurable lesions	
			Total population	Measurable	Non-measurable	(%)	Total
EORTC [37]	FAMTX	12	6.7	N/A	N/A	12.3 % (16/130)	12.8 % (50/390)
	ELF	9	7.2			14.8 % (19/128)	
	FUP	20	7.2			11.4 % (15/132)	
FLAGS [38]	CF	31.9	7.9	N/A	N/A	4.3 % (22/508)	4.2 % (43/1029)
	CS	29.1	8.6			4.0 % (21/521)	
V325 [39]	CF	25	8.6	N/A	N/A	N/A	N/A
	DCF	37	9.2			N/A	
CF versus IF [40]	CF	25.8	8.7	N/A	N/A	25.2 % (41/163)	24.3 % (81/333)
	IF	31.8	9.0			23.5 % (40/170)	
REAL-2 [41]	ECF	40.7	9.9	N/A	N/A	N/A	N/A
	ECX	46.4	9.9			N/A	
	EOF	42.4	9.3			N/A	
	EOX	47.9	11.2			N/A	
ML17032 [42]	FP	32	9.3	N/A	N/A	18.6 % (29/156)	18.7 % (59/316)
	XP	46	10.5			18.8 % (30/160)	

JCOG9912 [28] was the first pivotal trial to compare 5FU versus S-1 versus CPT11 plus cisplatin (CPT/CDDP) in Japan; however, it did not demonstrate the superiority of CPT/CDDP. As a result, S-1 was regarded as the standard regimen. S-1/CDDP was compared to S-1 monotherapy in the SPIRITS trial [3], which demonstrated the combination of cisplatin (60 mg/m^2^ for 8 days) with S-1 for 3 weeks on and 2-weeks off. The treatment was repeated every 5 weeks, unless disease progression was observed. The MST for S-1/CDDP and S-1 was 13.0 and 11.0 months, respectively, and the superiority of S-1/CDDP was demonstrated. A randomized phase III trial (TOP 002) was conducted to evaluate the safety and efficacy of IRIS (S-1/CPT-11) versus S-1 for advanced gastric cancer, which could not demonstrate a significant difference in patient survival [29]. The START trial was a randomized phase III study comparing S-1 alone with the S-1/docetaxel combination through the JACCRO GC03 trial [5]. This study was a prospective, multicenter, multinational (Korea and Japan), randomized, phase III study of patients with AGC. Docetaxel (40 mg/m^2^ on day 1) was administered with S-1 (80 mg/m^2^/day) for 2 weeks on and 1 week off. The MST for S-1/docetaxel and S-1 was 12.5 and 10.8 months, respectively, and the HR was 0.837 (95 % CI 0.711–0.985; *P* = 0.0319), showing the significant superiority of the doublet regimen. Moreover, in the patients with non-measurable lesions, MST for S-1/docetaxel and S-1 was 17.9 and 12.0 months, respectively. A prospective randomized control trial of adjuvant therapy for stage III GC is currently being conducted to compare S-1/docetaxel versus S-1 monotherapy as a control arm. On the other hand, as a modification of the 5-weekly S-1/CDDP regimen, the SOS trial demonstrated that the triweekly S-1/CDDP regimen has the same efficacy as the original schedule. In order to reduce the renal toxicity, oxaliplatin was used to replace cisplatin [30]. The G-SOX trial demonstrated an equal efficacy with lower toxicity and will be widely administered in general practice [31]. In the SOX regimen, oxaliplatin (100 mg/m^2^ on day 1) was administered with S-1 (80 mg/m^2^/day) for the first 2 weeks of a 3-week cycle.

The Trastuzumab for Gastric Cancer (ToGA) Study [7], a phase III trial of trastuzumab combined with standard chemotherapy, previously demonstrated an MST of 13.8 months for the trastuzumab plus chemotherapy arm compared to 11.1 months for the chemotherapy-alone arm (*P* = 0.0046) and showed significant improvements in time-to-progression (TTP) and progression-free survival (PFS) in the trastuzumab-treated group with a comparable toxicity profile. Capecitabine/5-FU and CDDP were the control arm schedule. The MSTs of XP in Japanese patients in the AVAGAST [32] and ToGA subset analysis [33] were 14.2 and 17.7 months, respectively. XP plus trastuzumab is regarded as the standard regimen in HER 2-positive patients as the first-level recommendation, and XP in HER 2-negative patients is the second-level recommendation.

More recently, DCS (Docetaxel/CDDP/S-1) therapy [34, 35] has been considered to be more effective and is currently being compared to S-1/CDDP in an ongoing randomized trial. Recent clinical trials have shown improved results in the treatment of stage IV gastric cancer. However, MST of stage IV GC remains from 3 to 4 months of best supportive care [36] to 17 months at most, which is summarized in Tables 1 and 2 [37–43], as explained above. Concerning the patients with non-measurable lesions including peritoneal disease, ascites, pleural effusion and minimal residual cancers, there can be a general tendency of longer survival compared to patients with measurable lesions according to subset analysis in most of the clinical trials. Tables 1 and 2 demonstrate the involvement of non-measurable lesions in the pivotal clinical trials in Japan and Western countries, respectively. In the START trial [5], as mentioned above, MST of S-1/docetaxel in non-measurable lesions was 17.9 M and that of S-1 alone was 12.0 M. Interestingly, MST of measurable lesions was not different between the two arms. Similar results were observed in the SPIRITS trial [3], which is regarded as the standard treatment in Japan, and more patients with non-measurable lesions were enrolled in the S-1/CDDP treatment group compared to the S-1 alone group in this study.

## Surgical intervention for stage IV GC

Gastric bypass, jejunojejunostomy, ileostomy and colostomy are sometimes performed for pylorus stenosis of the primary tumor and/or tumors of peritoneal disseminated disease in cases of unresectable GC. As is clarified in the Japanese guidelines, in many cases, even if an R0 resection cannot be achieved, the primary tumors are removed in palliative operations to correct bleeding and/or obstruction of the stomach and bowels [44–47]. In the case of pylorus stenosis, an expanding stent is now available [48]. The techniques for the use of the expanding stent have already been established and are becoming prevalent; however, they sometimes cause bleeding or dislocation. Recently, instead of using a bypass operation or metallic stenting, enteral nutritional management has often been performed, using a feeding tube over a pylorus ring in order to enhance nutrition and improve chemotherapy compliance as well as to administer oral fluoropyrimidine chemotherapy [49].

With regard to the palliative resection of the primary tumor, the REGATTA trial demonstrated that the initial removal of the primary tumor is not necessarily beneficial [50]. The indications for primary tumor resection in the trial were for stage IV GC patients who have only one affected organ other than the site of the primary tumor, such as peritoneal disease, several liver metastases or a paraaortic lymph node (LN) including 16a1 and 16b2.

According to the Japanese guidelines, the main strategy for the treatment of stage IV GC is palliative chemotherapy [2]. While the MST of stage IV GC has already been improved with the development of new chemotherapy regimens, it remains unsatisfactory. As mentioned above, we have previously demonstrated that the operative resection of the primary and/or metastatic lesions after successful chemotherapy can improve patient survival [21, 22]. Moreover, the survival of the patients with liver and/or lymph node metastasis had a better tendency compared to that with peritoneal dissemination [22]. Satoh et al. [23] demonstrated the feasibility of this approach with S-1 plus cisplatin, and Han et al. [24] demonstrated the survival benefit on curative resection in good responders to induction chemotherapy for patients with distant metastasis as well as peritoneal dissemination as reported by Okabe et al. [25]. However the patients who are eligible for such a procedure and the definition of the concept of “conversion therapy” remain to be clarified. The concept is currently the subject of confusion with regard to GC, because stage IV GC consists of heterogeneous conditions with a mixture of distant hematologic metastasis, distant LN metastasis and peritoneal dissemination. In the next section, we clarify the definitions of conversion therapy and suggest new categories of classification for stage IV GC based on oncosurgical treatment strategies.

## The new biological categories for the classification of stage IV GC

**Fig. 2:**
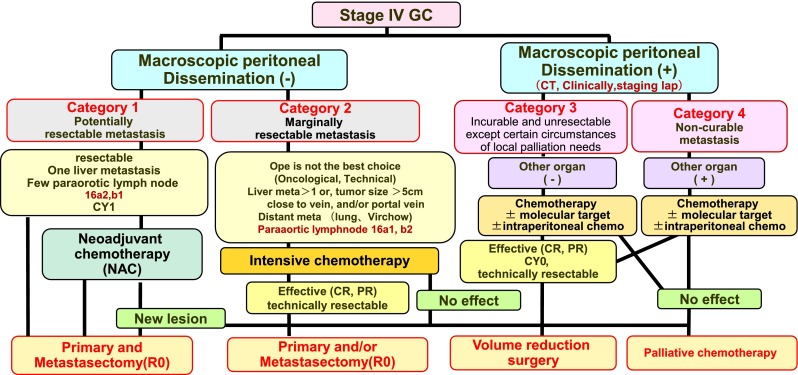
The new biological categories for the classification of stage IV GC

Figure 2 demonstrates the new biological categories for the classification of stage IV GC. We have divided this population into four categories. Initially, stage IV GC can be divided into two categories depending on the absence or presence of macroscopic peritoneal dissemination, which has a different biological outcome compared to hematological metastasis (by CT scan, PET-CT, barium enema, CT colonography, laparotomy or staging laparoscopy) [51, 52]. In the end, peritoneal dissemination causes the obstruction of bowels or malignant ascites, often leading to mechanical ileus and/or cachexia. However, the patients with hematologic metastasis such as liver, lung and other organs often die from organ failure. Moreover, it is quite difficult to remove the peritoneal dissemination completely because theoretically it is disseminated in whole abdominal cavity. On the other hand, measureable lesions in the organ deposit can be removed surgically if technically feasible. Patients without macroscopic peritoneal dissemination are further divided into category 1 (potentially resectable metastasis) and category 2 (marginally resectable metastasis) [53]. Patients who have macroscopic peritoneal dissemination are divided into category 3 (incurable and unresectable except certain circumstances of local palliation needs) and category 4 (noncurable metastasis). The patients with peritoneal disseminated disease diagnosed at the time of laparotomy or staging laparoscopy can be classified as category 3, even if they were not diagnosed by the routine diagnostic tools. Essentially all patients with peritoneal carcinomatosis from GC are not curable, irrespective of the pretreatment extent or the ability to achieve an R0 resection, but survival outcomes differ based on the degree of disease advancement and extent in addition to therapy response. The longer survival can be expected for patients corresponding to categories 1, 2 and, to a lesser extent, 3, while the treatment of other patients focuses on “care.”

This classification also address “whom can I operate on without induction chemotherapy” or “whom can I operate on after chemotherapy.” The concept of conversion therapy principally includes category 2, some category 3 patients and rarely category 4 patients when the operations are performed with the goal of an R0 resection or a surgical cure [18–20, 54].

### a) Category 1: Potentially resectable metastasis

This category includes patients with a single liver metastasis, with positive cytology or metastasis of the paraaortic LNs of no. 16a2 and/or 16b1. This category is regarded as an oncologically stage IV but technically resectable metastasis.

Regarding the cytologically positive GC patients, Kodera et al. [55] demonstrated that the 5-year survival of patients after gastrectomy without macroscopic peritoneal disease but with cytologically positive metastasis (only) is more than 20 %. This markedly improved survival was probably due to the S-1 adjuvant treatment. Moreover, the prognosis of patients with P0CY1 with R0 operation after chemotherapy is not yet known. Patients who were found P0 but CY1 can be included in this category. Whether such patients should be included in this category or another should be discussed with the accumulation of further cohort studies. As for liver metastasis, the survival of patients with a solitary liver metastasis was better than for patients with multiple metastases, suggesting the possible benefit of a metastasectomy at the time of primary tumor resection of the stomach [56–59]. Regarding paraaortic LN No. 16a2 and 16b1 metastasis, patients with these technically resectable metastases have rather good prognoses after treatment with neoadjuvant chemotherapy (NAC) with S-1/CDDP therapy according to the JCOG study [60–64]. Moreover, these cases were excluded from the REGATTA trial. The above-mentioned cases are considered to represent technically resectable metastasis; hence, primary tumor resection and metastasectomy can be considered with or without NAC. The term NAC is used to describe intensive chemotherapy in patients with tumors that are technically resectable [65–69]. Resection after successful chemotherapy, which achieves a range of responses [complete response (CR), partial response (PR) or even stable disease (SD)], may lead to better expected survival in this category in comparison to patients who are treated with chemotherapy alone. In patients in whom a new lesion occurs after NAC, palliative chemotherapy will be continued. In this category, patients can be treated with either an operation before chemotherapy or an operation after NAC irrespective of the response to the chemotherapy.

### b) Category 2: Marginally resectable metastasis

This category includes patients for whom the operation would not be considered to be the best choice for the initial treatment because of the presence of metastatic regions regarded as oncologically or technically unresectable. Regarding hepatic metastasis, patients with more than two liver metastases, a tumor size >5 cm or a tumor that is close to the hepatic and/or portal vein might be included in this category [50]. As for LN metastasis, paraaortic LN No. 16a1, 16b2 and distant LN metastasis, including the mediastinal, supraclavicular, axillary lymph nodes and distant organ metastasis, might also be included in this category. The patients in category 2 would be treated with first-line chemotherapy as the induction chemotherapy because it might achieve a good response in the regions targeted for resection and primary tumor resection might be performed when distant metastatic lesions are regarded as showing clinically complete response. There are patients with isolated distant metastases that turn out to be resectable. In this situation, most oncologists may treat the patient with chemotherapy, confirming that the tumor can respond to it or not, and/or at least no new lesions appear before resection, because this biological situation is accepted as generalized disease.

Conversion therapy can be defined as the surgical treatment (aiming at R0 resection) after chemotherapy of tumors that were originally regarded as technically or oncologically unresectable or only marginally resectable. The concept of the operation can be defined as conversion surgery or adjuvant surgery. Chemotherapy should be continued for as long as possible after removal of the tumors until the tumors acquire resistance to chemotherapy or until uncontrollable adverse events occur in the patients.

### c) Category 3: Incurable and unresectable except certain circumstances of local palliation needs

This category includes patients with only peritoneal disseminated disease detected by clinical routine examination as listed above, staging laparoscopy or at the time of initial open laparotomy. Recent chemotherapy can sometimes achieve the shrinking of bulky masses or peritoneal disseminated disease in the abdominal cavity [70]; however, it is quite rare for chemotherapy to extinguish all of the microscopic metastases, even after a satisfactory initial response, regardless of the presence or absence of metastasis in other organs [71–73]. When the metastatic site shows a good response to chemotherapy, primary tumors and/or metastatic tumors can be removed in the clinical setting after a staging laparoscopy confirming the CY0 and P0 situation. These operations can be defined as cytoreductive surgery or volume-reduction surgery even if a complete resection is performed because most of these cases recur afterwards in the peritoneal cavity [74]. Of course, this volume-reduction surgery can be partly included in the definition of conversion therapy. However, its clinical benefit should be clarified in the future.

In case of macroscopic peritoneal metastasis, even a single metastasis, limited to the lesser or greater omentum, this lesion might be included in this category. The lesion can be regarded as technically resectable but oncologically not.

### d) Category 4: Noncurable metastasis

Most of the GC patients with macroscopic peritoneal dissemination and other organ metastasis are regarded as unresectable or never resectable. Conversion therapy could only be considered in a small fraction of patients in whom exceptional response to the first-line chemotherapy rendered R0 resection possible. Otherwise, patients should continue to receive palliative chemotherapy. Of course, there can be some cases of the primary tumor resection in palliative operations to cope with bleeding and/or obstruction of the stomach and bowels with the option of a bypass operation.

## Discussion

We have previously demonstrated that surgical oncologists play a major role in conversion therapy or adjuvant surgery with chemotherapy regimens to further improve the prognosis of stage IV GC [21]. As reported elsewhere, this concept has recently received a great deal of attention. However, the definition of conversion therapy and the indications for operations remain to be clarified and, in the case of GC, are associated with a great deal of confusion. The reason for this is that previous reports included patients with GC who corresponded oncologically to stage IV but in whom metastasis was technically resectable. It has been previously demonstrated that such patients can be estimated to have better survival than patients with other metastatic lesions. Moreover, patients with peritoneal metastasis were also included in previous studies in which primary tumors were resected as cytoreductive surgery after successful induction chemotherapy. Long-term survival cannot be expected in such patients. Under these circumstances, it is not possible to clarify a significant role for conversion therapy.

In the present review, in order to better understand the biology and indications of curative surgery as a conversion therapy, we have proposed new categories for the classification of stage IV GC, taking into account the heterogeneous situation and treatment trends in general practice. In the new categories of classification, we have defined a potentially resectable metastasis as category 1, which can be resected technically but is oncologically considered stage IV. Such tumors can be technically resectable at the initial diagnosis and can be resected irrespective of their response to NAC unless new lesions appear. Such treatment is not considered to be “conversion therapy.”

The term “conversion therapy” describes a therapeutic concept in which the treatment strategy is converted by chemotherapy to curative surgery through an oncosurgical approach [21, 22, 75, 76]. The terms “conversion surgery” or “adjuvant surgery” can be applied to the operations performed for conversion therapy. Salvage surgery describes surgery performed to remove residual tumors or regrown tumors after curative radiation or chemoradiation therapy in cases where the tumor has invaded the adjacent organs, as is described in the Japanese guidelines for the treatment of esophageal cancer [77, 78]. The main difference is that salvage surgery is conducted in locally advanced tumors but conversion therapy is conducted in patients with metastatic lesions as well as primary tumor.

In the 1980s, the resection of primary tumors or the removal of metastatic disease was often conducted in the course of volume-reduction surgery. However, the prognosis of patients who underwent volume-reduction surgery was not considered to be satisfactory because the response rate to chemotherapy in those days was around 20–30 % [39]. It is only recently that the complete removal of tumors has been found frequently to be possible after successful treatment with S-1 based chemotherapy regimens. Recently, the REGATTA trial demonstrated that palliative surgery followed by chemotherapy in stage IV GC is not beneficial to categories 2 and 3 patients and that chemotherapy should be performed before primary tumor resection [50]. As we have demonstrated previously, there are two reasons for this: chemotherapy compliance tends to be better before gastrectomy, and the increased level of cytokines after surgery may enhance the proliferation of tumors [79, 80].

As mentioned above, the indications for conversion therapy include category 2 patients, some category 3 patients and category 4 patients in whom an R0 resection can be expected after a satisfactory response to chemotherapy. Primary tumor resections with an adequate LN dissection can be performed in cases when other distant metastases (such as peritoneal dissemination or distant lymph node, liver or lung metastases) are absent or when they respond completely to chemotherapy and when the complete removal of liver deposits is feasible (at a macroscopic level) and, moreover, when the minimal residual tumors in distant LN metastasis after chemotherapy can be extensively removed. Several issues should be discussed in order to clarify this concept, including the timing of the operation, whether R0 operations are required, the best treatment regimen and whether chemotherapy is required after an R0 operation.

The best timing for the operation is generally when the tumor displays the best response to chemotherapy (not when the tumor is increasing in size or when it has acquired the ability to regrow). This has been demonstrated elsewhere in GIST treatment [81, 82]. Generally, we estimate the best timing for the removal of the tumor to be when a CR or PR response is detected during the performance of 4–6 cycles of S-1/CDDP or S-1/docetaxel regimens. However, as Yoshikawa et al. [83] reported recently, in the case of NAC, two cycles might be sufficient. Of course, the continuation of chemotherapy after such surgery might be required until the tumor acquires resistance to chemotherapy or uncontrollable adverse events occur, even after R0 resections [21, 84–86].

In conclusion, the administration of conversion therapy for stage IV GC cases might be one of the main roles of the surgical oncologist in the near future. This is a new therapeutic concept that warrants clinical evaluation by a prospective cohort study and/or randomized control trial. We are now conducting a prospective cohort study in order to estimate the feasibility of this concept (UMIN-ID: 000004787, https://upload.umin.ac.jp/cgi-open-bin/ctr/ctr.cgi?function=search&action=input). Moreover, large-scale retrospective and prospective cohort studies are currently being conducted in Asia through the Federation of Asian Clinical Oncology (FACO), which consists of the Japanese Society of Clinical Oncology (JSCO), Korean Association of Clinical Oncology (KACO) and Chinese Society of Clinical Oncology (CSCO), with the support of the Japanese Gastric Cancer Association (JGCA), Korean Gastric Cancer Association (KGCA) and Gastric Cancer Association of the Chinese Anti-cancer Association.
